# Clinical outcome of bevacizumab or ramucirumab combined with epidermal growth factor receptor (EGFR) tyrosine kinase inhibitors as the first line therapy in susceptible EGFR‐mutated advanced non‐small‐cell lung

**DOI:** 10.1002/kjm2.12822

**Published:** 2024-03-25

**Authors:** Chia‐Yu Kuo, Ming‐Ju Tsai, Jen‐Yu Hung, Mei‐Hsuan Lee, Kuan‐Li Wu, Yu‐Chen Tsai, Cheng‐Hao Chuang, Chung‐Wen Huang, Chin‐Ling Chen, Chih‐Jen Yang, Inn‐Wen Chong

**Affiliations:** ^1^ Division of Pulmonary and Critical Care Medicine, Department of Internal Medicine Kaohsiung Medical University Hospital, Kaohsiung Medical University Kaohsiung Taiwan; ^2^ Division of Pulmonary and Critical Care Medicine, Department of Internal Medicine Kaohsiung Municipal Siaogang Hospital Kaohsiung Taiwan; ^3^ School of Medicine, College of Medicine Kaohsiung Medical University Kaohsiung Taiwan; ^4^ School of Post‐Baccalaureate Medicine College of Medicine, Kaohsiung Medical University Kaohsiung Taiwan; ^5^ Department of Internal Medicine Kaohsiung Municipal Ta‐Tung Hospital, Kaohsiung Medical University Kaohsiung Taiwan; ^6^ Cancer Center Kaohsiung Medical University Hospital, Kaohsiung Medical University Kaohsiung Taiwan; ^7^ Department of Respiratory Therapy College of Medicine, Kaohsiung Medical University Kaohsiung Taiwan

**Keywords:** epidermal growth factor receptor, lung adenocarcinoma, tyrosine kinase inhibitor, vascular endothelial growth factor

## Abstract

Combining epidermal growth factor receptor (EGFR) tyrosine kinase inhibitor (TKI) with an anti‐ vascular endothelial growth factor (VEGF) agent, bevacizumab or ramucirumab, is indicated for advanced lung adenocarcinoma harboring EGFR mutation. This study aimed to show the real‐world data of combination therapy and compare the effectiveness between bevacizumab and ramucirumab in combination with an EGFR‐TKI. This retrospective study enrolled 47 patients diagnosed of stage IV lung adenocarcinoma with exon 19 deletion or L858R point mutation, receiving a first‐line EGFR‐TKI with anti‐VEGF agent, including 34 (72%) and 13 (28%) patients receiving bevacizumab and ramucirumab, respectively. The response rate was similar in both groups (*p* = 0.38). Patients receiving bevacizumab had similar progression free survival (PFS) as those receiving ramucirumab (median PFS: 21.9 vs. 24.2 months, *p* = 0.4871); similar finding was noted in overall survival (OS) (median OS: 33.5 months vs. not reached, *p* = 0.4618). Patients receiving ramucirumab experienced a significantly high‐grade hypertension compared to those receiving bevacizumab (*p* = 0.0351). Multivariable Cox regression analysis found independent risk factors for worse PFS included poorer ECOG performance status, multiple (≥3) metastatic sites, brain metastasis, and pleural metastasis/effusion, while the type of anti‐VEGF agent was not a risk factor. Pericardial metastasis/effusion was the only one independent risk factor for worse OS. In summary, ramucirumab may have similar effectiveness as bevacizumab in combination with an EGFR‐TKI as first line therapy for advanced lung adenocarcinoma harboring susceptible EGFR mutation. Further large‐scale registry‐based cohort studies may be needed to validate our findings.

## 1 INTRODUCTION

Non‐small cell lung cancer (NSCLC) is the most common cause of cancer‐related mortality worldwide, among which the most common cell type is adenocarcinoma.^1^ Earlier, the standard treatment for NSCLC patients was chemotherapy.^2^ Since the development of epidermal growth factor receptor (EGFR) tyrosine kinase inhibitors (TKIs), clinical practice has changed. The incidence rate of EGFR mutation in NSCLC patients is approximately 40%–60% in Asian individuals and 10% in Western individuals.^3^ In recent years, several phase III randomized trials have further reported that patients with NSCLC harboring EGFR mutations, such as exon 21 L858R point mutation and exon 19 deletion, have better progression‐free survival (PFS) when treated with an EGFR TKI.^4^, ^5^, ^6^, ^7^, ^8^, ^9^, ^10^, ^11^ Despite the good initial response, acquired resistance almost always develops earlier or later. The main resistant mechanism of first‐ and second‐generation EGFR‐TKIs is the development of T790M mutation.^12^

The vascular endothelial growth factor (VEGF) pathway is well established as one of the key regulators of angiogenesis in the tumor microenvironment.^13^ Activation of the VEGF‐receptor pathway triggers a network of signaling processes that promote endothelial cell growth and migration, as well as vascular permeability, which has been associated with malignant effusions.^14^ Two anti‐VEGF agents, bevacizumab and ramucirumab, have been approved for the treatment of advanced NSCLC by the United States *Food and Drug Administration (US FDA)*.^15^ Recent research suggests that combining anti‐VEGF agents with chemotherapy may offer a more effective treatment approach for advanced non‐squamous NSCLC, compared to using chemotherapy by itself.^16^, ^17^ In REVEL study, second‐line treatment with ramucirumab plus docetaxel improved the Progression free survival (PFS) and overall survival (OS) of patients with advanced NSCLC.^18^

Recent randomized controlled trials have shown that treatment with an EGFR‐TKI combined with an anti‐VEGF agent, comparted to using an EGFR‐TKI alone, provided better PFS, especially in patients with exon 21 L858R. In JO25567 and NEJ026 studies, combination therapy with bevacizumab and erlotinib improves PFS, compared with using erlotinib alone, in patients with EGFR‐positive NSCLC, especially for those having pleural/pericardial effusion.^19^, ^20^ In RELAY study, ramucirumab plus erlotinib demonstrated superior PFS compared with erlotinib alone in patients with untreated EGFR‐mutated metastatic NSCLC.^21^ In addition, the efficacy of combining an anti‐VEGF agent and immunotherapy for advanced NSCLC has been demonstrated as well.^22^, ^23^

Bevacizumab and ramucirumab play a similar role to stop tumor angiogenesis. However, their efficacy has not been compared in the treatment of advanced NSCLC. This real‐world study aimed to compare the effectiveness and safety of first‐line bevacizumab and ramucirumab in patients of advanced EGFR‐mutant adenocarcinoma receiving a first‐line EGFR TKI.

## 2 PATIENTS AND METHODS

### 2.1 Patient identification

**FIGURE 1:**
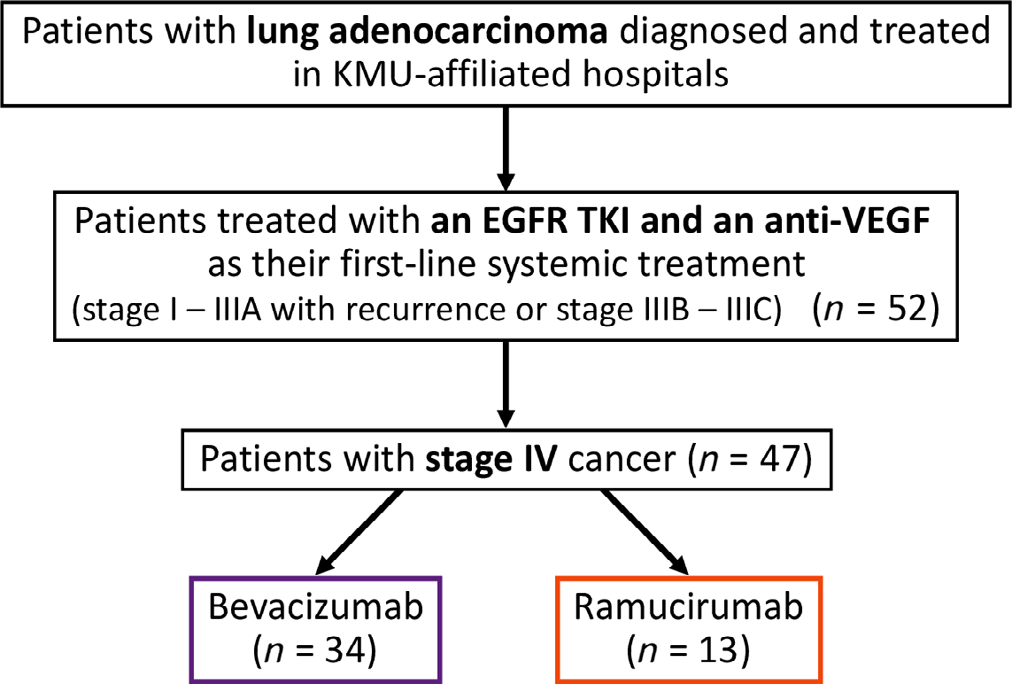
Flowchart for identifying the study population. EGFR, epidermal growth factor receptor; KMU, Kaohsiung Medical University; VEGF, vascular endothelial growth factor.

We enrolled patients with lung adenocarcinoma diagnosed and treated in three Kaohsiung Medical University (KMU)‐affiliated hospitals, including Kaohsiung Medical University Hospital (KMUH), Kaohsiung Municipal Ta‐Tung Hospital, and Kaohsiung Municipal Siaogang Hospital (Figure 1). The diagnosis of lung adenocarcinoma was confirmed pathologically according to the World Health Organization pathology classification, and cancer staging was confirmed by lung cancer teams according to the 8th version of staging system by American Joint Committee on Cancer. The genomic DNA, extracted from the tissue block, was subject to genotyping of exons 18–21 of the EGFR gene using real‐time polymerase chain reaction (PCR) (cobas EGFR Mutation Test v2). The kit is a ready‐to‐use kit for the detection of 42 somatic mutations in the EGFR cancer‐related gene using PCR on the cobas z480 instrument and cobas 4800 analyzer. The examination techniques were consistent with our previous studies.^24^, ^25^, ^26^, ^27^, ^28^, ^29^ The Institutional Review Board (IRB) of Kaohsiung Medical University Hospital (KMUH) approved this study (KMUHIRB‐E(I)‐20210257) and waived the need for written informed consent from all patients.

In the current study, we enrolled all individuals of lung adenocarcinoma with only exon 19 deletion or exon 21 L858R point mutation and patients were all naïve to systemic treatment and were treated with an EGFR TKI plus an anti‐VEGF agent as their first‐line systemic treatment. The EGFR TKIs included first generation (gefitinib and erlotinib), second generation (afatinib and dacomitinib), and third generation (osimertinib). The anti‐VEGF agents included bevacizumab and ramucirumab. Patients received bevacizumab at a dose of 7.5 mg/kg body weight every 3 weeks. The dose of ramucirumab was 10 mg/kg body weight every 2–3 weeks. Baseline clinical characteristics were determined by retrospective review of medical records, including age at diagnosis, sex, Eastern Cooperative Oncology Group (ECOG) performance status (PS), EGFR mutation, programmed cell death‐ligand 1 (PD‐L1), site of metastasis and the type of EGFR TKI.

The initial treatment response was classified based on serial imaging studies using the revised Response Evaluation Criteria in Solid Tumors (RECIST 1.1) criteria. The PFS and OS were defined as the time from the initiation of the EGFR TKI to the date of disease progression on an imaging examination and the date of death, respectively.

### 2.2 Statistical analysis

Categorical variables and continuous variables were compared using Fisher's exact test and Wilcoxon rank‐sum test, respectively. Survival times were estimated using Kaplan–Meier method, and differences between groups were compared with log‐rank test. Both univariate and multivariable Cox regression analyses were used to determine the predictive factors for PFS and OS, and hazard ratios (HR) with 95% confidence intervals (CIs) are presented. All statistical analyses were performed with SAS software (version 9.4 for Windows, SAS Institute Inc., Cary, NC, USA). Statistical significance was set at a two‐tailed *p* value of <0.05.

## 3 RESULTS

We identified 52 patients of lung adenocarcinoma treated with an EGFR TKI plus an anti‐VEGF agent as their first‐line systemic treatment (Figure 1). After excluding those with stage I–IIIA with recurrence or stage IIIB–IIIC disease, the remaining 47 patients with stage IV disease were enrolled for further analyses (Table 1). The median (interquartile range) age of enrolled patients was 62.3 (57.0–71.5) years, and 11 (23%) patients were male. Most of our patients (91%) were never smokers. Most of the patients had a good performance status (89% of them had ECOG PS ≤ 1) on diagnosis. Exon 19 deletion and exon 21 L858R were detected in the tumors of 18 (38%) and 30 (64%) patients, respectively (a patient's tumor had both mutation). PD‐L1 test by immunohistochemistry was done in 31 patients, including 16 patients having PD‐L1 ≥ 1% and 15 patients having PD‐L1 < 1%.

**TABLE 1:** Baseline characteristics of the study cohort.

Variables	All patients	Bevacizumab	Ramucirumab	*p* Value
*n*	47	34	13	
Sex				
Female	36 (77%)	28 (82%)	8 (62%)	0.2461
Male	11 (23%)	6 (18%)	5 (38%)	
Age (year)	62.3 (57.0–71.5)	66.4 (59.1–72.7)	57.0 (52.7–58.7)	0.0014
<65	27 (57%)	15 (44%)	12 (92%)	0.0031
≥65	20 (43%)	19 (56%)	1 (8%)	
Smoking history				
Never	43 (91%)	31 (91%)	12 (92%)	0.7400
Current smoker	2 (4%)	2 (6%)	0 (0%)	
Ex‐smoker	2 (4%)	1 (3%)	1 (8%)	
ECOG performance status				
≤1	42 (89%)	29 (85%)	13 (100%)	0.3027
≥2	5 (11%)	5 (15%)	0 (0%)	
EGFR mutation				
Exon 19 deletion	18 (38%)	11 (32%)	7 (54%)	0.1986
Exon 21 L858R	30 (64%)	24 (71%)	6 (46%)	0.1762
PD‐L1^a^				
<1%	15 (48%)	9 (43%)	6 (60%)	0.4578
≥1%	16 (52%)	12 (57%)	4 (40%)	
Metastatic sites				
<3	27 (57%)	21 (62%)	6 (46%)	0.5107
≥3	20 (43%)	13 (38%)	7 (54%)	
Metastasis				
Brain/leptomeningeal metastasis	22 (47%)	13 (38%)	9 (69%)	0.1009
Lung metastasis	28 (60%)	21 (62%)	7 (54%)	0.7431
Pleural metastasis/effusion	24 (51%)	18 (53%)	6 (46%)	0.7516
Bone metastasis	25 (53%)	17 (50%)	8 (62%)	0.5303
Liver metastasis	7 (15%)	4 (12%)	3 (23%)	0.3769
Pericardial metastasis	3 (6%)	2 (6%)	1 (8%)	>0.99
Adrenal metastasis	1 (2%)	1 (3%)	0 (0%)	>0.99
Other metastasis	5 (11%)	2 (6%)	3 (23%)	0.1213
EGFR TKI				
Gefitinib	5 (11%)	5 (15%)	0 (0%)	0.2138
Erlotinib	22 (47%)	14 (41%)	8 (62%)	
Afatinib	18 (38%)	14 (41%)	4 (31%)	
Dacomitinib	1 (2%)	1 (3%)	0 (0%)	
Osimertinib	1 (2%)	0 (0%)	1 (8%)	
Operation ^b^ ^,^ ^c^	7 (15%)	2 (6%)^b^	5 (38%)^c^	0.0124

*Note*: Data are presented in *n* (%) or median (interquartile range). *p* Values were assessed with Fisher's exact test or Wilcoxon rank‐sum test.

Abbreviations: ECOG, Eastern Cooperative Oncology Group; EGFR, epidermal growth factor receptor; PD‐L1, programmed death ligand 1; TKI, tyrosine kinase inhibitor.

^a^ PD‐L1 were examined in 31 patients.

^b^ One patient underwent salvage wedge resection and another underwent lobectomy on disease progression.

^c^ One patient underwent lobectomy for tissue proof prior to treatment, two patients underwent salvage segmentectomy, and two patients underwent salvage lobectomy.

In the enrolled 47 patients, 20 (43%) patients had multiple (≥3) metastatic sites; 22 (47%), 28 (60%), 24 (51%), and 25 (53%) patients had metastasis to brain/leptomeninges, lung, pleura (or pleural effusion), and bone, respectively. Most of the patients received erlotinib (47%) and afatinib (38%) as their first‐line EGFR TKI.

**FIGURE 2:**
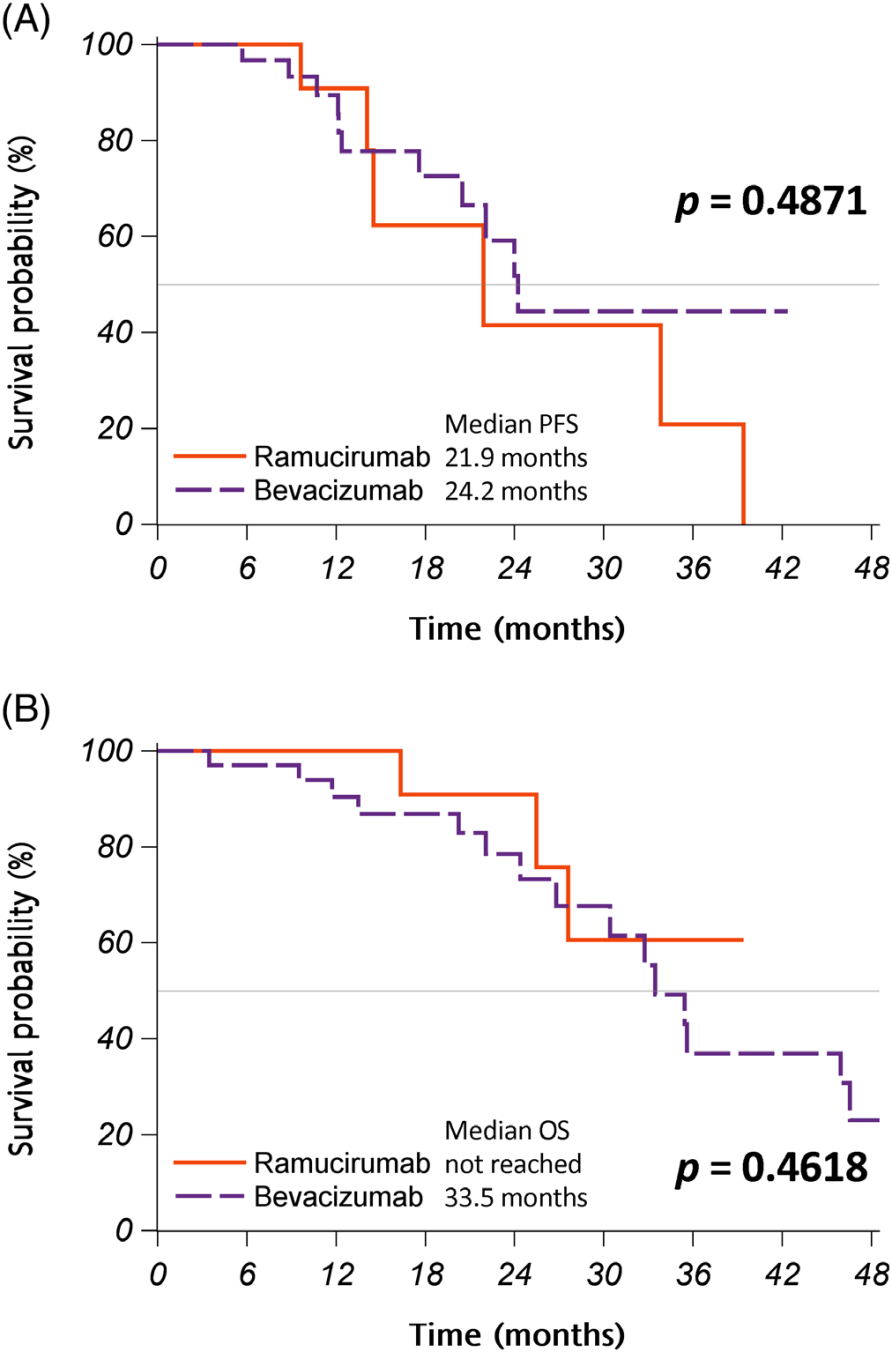
The progression‐free survival (PFS) (A) and overall survival (OS) (B) of patients.

In terms of the anti‐VEGF agent, 34 (72%) and 13 (28%) patients received bevacizumab and ramucirumab, respectively (Table 1). Patients received different anti‐VEGF agents had similar smoking history and performance status. It appeared that elder patients were less likely to be treated with ramucirumab (*p* = 0.0031); patients receiving bevacizumab were significantly older than those receiving ramucirumab (median age: 66.4 vs. 57.0 years, *p* = 0.0014). The response rate (88% vs. 77%, *p* = 0.3769) and disease control rate (100% vs. 100%, *p* > 0.99) were similar in both groups (Table 2). Patients treated with ramucirumab experienced a significantly higher‐grade hypertension than those treated with bevacizumab (*p* = 0.0351), despite the overall rates of hypertension were similar in both groups (*p* = 0.3262). The incidence rates of other adverse events were similar in both groups. The most common recurrent sites were lung (38%) and brain/leptomeninges (21%), and patients receiving different anti‐VEGF agents showed similar incidences in terms of recurrent sites. Re‐biopsy for T790M detection, either a liquid or tissue biopsy, after disease progression of first line therapy was performed in 30 patients, while there was no significant difference in T790M detection rate between the patients receiving bevacizumab and ramucirumab (32% vs. 63%, *p* = 0.2098). Patients receiving bevacizumab had similar PFS as those receiving ramucirumab (median PFS: 24.2 vs. 21.9 months, *p* = 0.4871) (Figure 2A); similar finding was also noted in OS (median OS: 33.5 months vs. not reached, *p* = 0.4618) (Figure 2B).

**TABLE 2:** Treatment responses, adverse events, recurrent sites, and subsequent T790M mutation.

Variables	All patients (%)	Bevacizumab (%)	Ramucirumab (%)	*p* Value
Initial treatment response^a^				
Stable disease	7 (15)	4 (12)	3 (23)	0.3769
Partial response	40 (85)	30 (88)	10 (77)	
Disease control rate	47 (100)	34 (100)	13 (100)	>0.99
Response rate	40 (85)	30 (88)	10 (77)	0.3769
Adverse event (any)				
Hypertension	6 (13)	3 (9)	3 (23)	0.3262
Proteinuria	5 (11)	3 (9)	2 (15)	0.6070
Bleeding	9 (19)	6 (18)	3 (23)	0.6921
Others	3 (6)	3 (9)	0 (0)	0.5502
Adverse event				
Hypertension				
Grade 0	41 (87)	31 (91)	10 (77)	0.0351
Grade 1	3 (6)	3 (9)	0 (0)	
Grade 2	3 (6)	0 (0)	3 (23)	
Proteinuria				
Grade 0	42 (89)	31 (91)	11 (85)	0.6070
Grade 1	5 (11)	3 (9)	2 (15)	
Bleeding				
Grade 0	38 (81)	28 (82)	10 (77)	0.6508
Grade 1	3 (6)	1 (3)	2 (15)	
Grade 2	4 (9)	3 (9)	1 (8)	
Grade 3	1 (2)	1 (3)	0 (0)	
Grade 4	1 (2)	1 (3)	0 (0)	
Others				
Grade 0	44 (94)	31 (91)	13 (100)	0.5502
Grade 1	3 (6)	3 (9)	0 (0)	
Recurrent site				
Brain/leptomeningeal	10 (21)	6 (18)	4 (31)	0.4294
Lung	18 (38)	14 (41)	4 (31)	0.7386
Pleura / pleural effusion	0 (0)	0 (0)	0 (0)	>0.99
Bone	3 (6)	1 (3)	2 (15)	0.1812
Liver	3 (6)	2 (6)	1 (8)	>0.99
Adrenal gland	0 (0)	0 (0)	0 (0)	>0.99
Other	0 (0)	0 (0)	0 (0)	>0.99
T790M results				
Not detected	18 (60)	15 (68)	3 (38)	0.2098
Detected (either tissue or liquid)	12 (40)	7 (32)	5 (63)	

*Note*: Data are presented in *n* (%). *p* Values were assessed with Fisher's exact test.

^a^ Initial treatment response were assessed in 47 patients.

Cox regression analyses were used to identify the risk factors associated with poorer PFS and OS. Univariate analyses revealed that multiple (≥3) metastatic sites and brain metastasis was significantly associated with poorer PFS (Table 3). Multivariable analysis showed independent risk factors for poorer PFS included worse PS (ECOG PS ≥ 2) (HR [95%CI]: 6.36 [1.10–36.75] in model 2R), multiple (≥3) metastatic sites (3.05 [1.08–8.60] in model 1R), brain metastasis (3.96 [1.28–12.26] in model 2R), and pleural metastasis/effusion (3.69 [1.13–12.07] in model 2R), while the type of anti‐VEGF was not associated with better or worse PFS. In terms of OS, pericardial metastasis/effusion was the only factors associated with poorer OS in univariate analyses brain metastasis (Table 4). Multivariable Cox regression analyses identified independent risk factors for poorer OS included pericardial metastasis/effusion (7.72 [1.36–43.69] in model 4R), while the type of anti‐VEGF was not associated with better or worse OS.

**TABLE 3:** COX regression to identify factors associated with progression free survival.

Variables	Univariate	Multivariable model 1	Multivariable model 1R	Multivariable model 2	Multivariable model 2R
Sex (male vs. female)	1.12 [0.36–3.54]	0.90 [0.22–3.57]		3.46 [0.29–41.05]	
Age (≥65 vs. <65)	0.42 [0.15–1.15]	0.45 [0.13–1.62]		33.37 [0.47–2362.18]	
ECOG PS (≥2 vs. ≤1)	3.14 [0.65–15.29]	7.21 [1.13–46.18]*		0.16 [0.00–18.48]	6.36 [1.10–36.75]*
Deletion in exon 19 (yes vs. no)	0.92 [0.35–2.42]	0.45 [0.10–2.03]		0.13 [0.02–0.90]*	
Operation (yes vs. no)	0.45 [0.06–3.45]	0.50 [0.05–4.69]		^a^	
EGFR TKI (2nd or 3rd generation vs. 1st generation)	1.24 [0.48–3.22]	2.85 [0.73–11.23]		3.87 [0.65–22.93]	2.71 [0.85–8.59]
Anti‐VEGF (ramucirumab vs. bevacizumab)	1.44 [0.51–4.03]	1.61 [0.31–8.28]		1.65 [0.19–14.56]	
Metastatic sites (≥3 vs. ≤2)	3.05 [1.08–8.60]*	3.96 [1.24–12.67]*	3.05 [1.08–8.60]*		
Brain metastasis (vs. no)	3.05 [1.06–8.71]*			7.68 [0.74–79.53]	3.96 [1.28–12.26]*
Lung metastasis (vs. no)	1.48 [0.51–4.33]			34.79 [1.15–1049.80]*	
Bone metastasis (vs. no)	1.22 [0.45–3.30]			127.77 [2.22–7346.40]*	
Pleural metastasis/effusion (vs. no)	2.48 [0.86–7.13]			5.03 [0.90–28.25]	3.69 [1.13–12.07]*
Liver metastasis (vs. no)	0.00 [0.00–.]			^a^	
Pericardial metastasis/effusion (vs. no)	3.88 [0.84–17.91]			^a^	
Adrenal metastasis (vs. no)	1.28 [0.16–9.91]			0.07 [0.00–1.64]	
Other metastasis (vs. no)	2.16 [0.60–7.77]			24.01 [1.40–411.01]*	

*Note*: A patient with cancer harboring both exon 19 deletion and L858R was classified to the exon 19 deletion group. Data are presented in hazard ratio (HR) [95% confidence interval]. After building the maximal models of multivariable Cox regression (models 1 and 2), the corresponding reduced models (models 1R and 2R, respectively) were built using backward variable selection method, keeping only variables with *p* values < 0.1.

Abbreviations: ECOG, Eastern Cooperative Oncology Group; EGFR, epidermal growth factor receptor; PS, performance status; TKI, tyrosine kinase inhibitor; VEGF, vascular endothelial growth factor.

^a^ HR cannot be assessed due to small sample size.

* *p* Value < 0.05.

**TABLE 4:** COX regression to identify factors associated with overall survival.

Variables	Univariate	Multivariable model 3	Multivariable model 3R	Multivariable model 4	Multivariable model 4R
Sex (male vs. female)	2.28 [0.86–6.01]	2.49 [0.74–8.43]	2.28 [0.86–6.01]	8.01 [1.51–42.44]*	2.30 [0.86–6.15]
Age (≥65 vs. <65)	1.05 [0.43–2.55]	0.78 [0.25–2.45]		2.76 [0.63–12.03]	
ECOG PS (≥2 vs. ≤1)	1.92 [0.54–6.79]	1.63 [0.35–7.59]		0.21 [0.03–1.71]	
Deletion in exon 19 (yes vs. no)	0.88 [0.33–2.33]	1.07 [0.25–4.51]		1.05 [0.22–5.03]	
Operation (yes vs. no)	^a^	^a^		^a^	
EGFR TKI (2nd or 3rd generation vs. 1st generation)	1.41 [0.54–3.68]	1.24 [0.33–4.56]		0.64 [0.11–3.80]	
Anti‐VEGF (ramucirumab vs. bevacizumab)	0.63 [0.18–2.20]	0.52 [0.11–2.55]		0.97 [0.11–8.72]	
Metastatic sites (≥3 vs. ≤2)	1.02 [0.38–2.72]	1.49 [0.46–4.82]			
Brain metastasis (vs. no)	0.76 [0.26–2.19]			1.21 [0.20–7.29]	
Lung metastasis (vs. no)	0.76 [0.31–1.86]			0.95 [0.25–3.67]	
Bone metastasis (vs. no)	1.93 [0.73–5.13]			5.12 [1.07–24.47]*	2.50 [0.87–7.14]
Pleural metastasis/effusion (vs. no)	1.19 [0.48–2.94]			4.00 [0.72–22.14]	
Liver metastasis (vs. no)	0.92 [0.29–2.90]			0.32 [0.04–2.38]	
Pericardial metastasis/effusion (vs. no)	5.23 [1.05–26.07]*			64.88 [2.23–1889.10]*	7.72 [1.36–43.69]*
Adrenal metastasis (vs. no)	^a^			^a^	
Other metastasis (vs. no)	^a^			^a^	

*Note*: A patient with cancer harboring both exon 19 deletion and L858R was classified to the exon 19 deletion group. Data are presented in hazard ratio (HR) [95% confidence interval]. After building the maximal models of multivariable Cox regression (models 3 and 4), the corresponding reduced models (models 3R and 4R, respectively) were built using backward variable selection method, keeping only variables with *p* values < 0.1.

Abbreviations: ECOG, Eastern Cooperative Oncology Group; EGFR, epidermal growth factor receptor; PS, performance status; TKI, tyrosine kinase inhibitor; VEGF, vascular endothelial growth factor.

^a^ HR cannot be assessed due to small sample size.

* *p* Value < 0.05.

## 4 DISCUSSION

This study demonstrated the real‐world data about the combination of an EGFR TKI and an anti‐VEGF agent as the first‐line therapy for stage IV lung adenocarcinoma harboring susceptible EGFR mutation in Taiwan. In those receiving a first‐line EGFR TKI, patients receiving add‐on bevacizumab and ramucirumab had similar initial treatment response, PFS, and OS, as well as the T790M detection rate on disease progression. Patients treated with ramucirumab experienced a significantly high‐grade hypertension than those treated with bevacizumab. Worse PS (ECOG PS ≥ 2), multiple (≥3) metastatic sites, brain metastasis, and pleural metastasis/effusion were independent risk factor for worse PFS, while the type of anti‐VEGF did not affect PFS. Pericardial metastasis/effusion was the only one independent risk factor for worse OS identified by Cox regression models.

Generally speaking, the median PFS of advanced lung adenocarcinoma treated with an EGFR TKI is about 9–13 months as shown in several phase III randomized controlled trials.^4^, ^5^, ^6^, ^7^, ^8^, ^9^, ^10^, ^11^ Although remaining challenging, several strategies have been tried to prolong the PFS, including add‐on anti‐VEGF agent and local consolidative therapy.^29^ In NEJ026 study, a randomized, open‐label, phase 3 trial comparing erlotinib plus bevacizumab combination therapy with erlotinib monotherapy, the combination group showed improved PFS (median PFS: 16.9 vs. 13.3 months; HR [95%CI]: 0.61 [0.42–0.88], *p* = 0.016).^19^ In a real‐world study evaluating the effectiveness of combination therapy with an EGFR‐TKI plus bevacizumab versus EGFR‐TKI monotherapy for patients with EGFR‐mutated advanced NSCLC, combination therapy showed significantly longer PFS (median PFS: 17.0 vs. 11.0 months; HR [95%CI]: 0.48 [0.30–0.77], *p* = 0.002).^30^ Another real‐world study of advanced EGFR‐mutated NSCLC showed that combination therapy with an first‐line EGFR‐TKI and bevacizumab resulted in the objective response rate of 77.8%, disease control rate of 94.4%, and median PFS of 16.4 months; in terms of the type of EGFR TKI, patients taking erlotinib had similar PFS compared to those taking afatinib (median PFS: 17.1 vs. 21.6 months, *p* = 0.217).^31^ In our study, the median PFS was 24.2 months and the response rate was 88% in the group of the patients receiving an EGFR TKI plus bevacizumab. In the RELAY study, a worldwide, double‐blind, phase 3 trial assessing erlotinib plus ramucirumab or placebo in patients with untreated EGFR‐mutated metastatic NSCLC, the combination group showed significantly better PFS (median PFS: 19.4 vs. 12.4 months, *p* < 0.001) and a good response rate (76%).^21^ Similarly, our study revealed that patients receiving an EGFR TKI plus ramucirumab had a median PFS of 21.9 months and response rate of 77%, while the EGFR TKI was not limited to erlotinib.

Ramucirumab acted on the similar pathway as bevacizumab.^15^ Till now, there was no prospective clinical study directly comparing the clinical outcomes between bevacizumab and ramucirumab as an add‐on treatment to an EGFR TKI for EGFR‐mutated advanced NSCLC. In a recent retrospective real‐world study enrolling 96 patients to compare the clinical outcomes between bevacizumab and ramucirumab as an add‐on treatment to an EGFR TKI, erlotinib (44.8%) and afatinib (37.5%) were the most commonly used EGFR TKIs, while 42 patients received an anti‐VEGF agent (23 and 19 patients used bevacizumab and ramucirumab, respectively) as the front‐line therapy.^32^ Comparing the patients receiving add‐on bevacizumab and ramucirumab as the front‐line therapy, there was no significant difference in the PFS (median PFS: 24.1 vs. 15.7 months, *p* = 0.454) and OS (median OS: 48.6 vs. 43.0 months, *p* = 0.924). Multivariable analysis revealed that receiving eight or more cycles of the anti‐VEGF agent was an independent factor for better OS (HR [95% CI]: 0.45 [0.24–0.85]; *p* = 0.014).^32^ In line with their findings, our study of patients receiving an anti‐VEGF agent as an add‐on treatment to the first‐line EGFR TKI also demonstrated similar PFS (median PFS: 24.2 vs. 21.9 months) and OS (median OS: 33.5 months vs. not reached) in bevacizumab and ramucirumab users. We also found that worse PS and multiple (≥3) metastatic sites were independent prognostic factors for poorer PFS, while the type of anti‐VEGF agent was not.

T790M mutation is the common on‐target mutation leading to drug resistance in EGFR‐TKI‐treated NSCLC patients, and may be a favorable prognostic factor for both OS and PFS. Indeed, patients of lung adenocarcinoma with acquired T790M‐mutation showed a favorable outcome while treated with osimertinib than with standard platinum‐based chemotherapy.^33^ The incidence rate for T790M mutation after disease progression of a first‐line EGFR TKI is approximately 50%.^34^, ^35^ In the RELAY study, the frequency of treatment‐emergent EGFR T790M was similar between the patients receiving erlotinib plus ramucirumab and erlotinib alone (43% vs. 47%, *p* = 0.849).^21^ In the TERRA study, a retrospective, multicenter study conducted in Taiwan which evaluated the T790M detection rate after a first‐line combination therapy with bevacizumab and an EGFR‐TKI in advanced NSCLC, the incidence rate of T790M after acquired resistance was 55.1%.^36^ In another real‐world study analyzing the clinical outcomes of NSCLC patients with brain metastasis, the T790M‐positive rate did not differ significantly between patients using an EGFR‐TKI plus bevacizumab and those using EGFR‐TKI monotherapy (66.7% vs. 75.0%, *p* = 0.460).^37^ The incidence of T790M mutation was not influenced by adding different anti‐VEGF agent. In a recent study evaluating the clinical outcomes of different anti‐VEGF agents as an add‐on therapy to an EGFR TKI, the rates of T790M detection were similar between patients using bevacizumab and ramucirumab (43.6% vs. 38.2%, *p* = 0.645).^32^ In line with the previous study, our study showed no significant difference in T790M detection rate between patients receiving add‐on bevacizumab and ramucirumab (32% vs. 63%) while only 64% of patients had T790M testing on disease progression.

Bevacizumab and ramucirumab had similar safety profiles. The most common adverse effect associated with anti‐VEGF agents included hypertension, bleeding, and proteinuria.^19^, ^20^, ^21^ In the present study, the incidences of adverse events were generally similar between bevacizumab and ramucirumab, except for significantly high‐grade hypertension in ramucirumab users than bevacizumab users.

Our study had several limitations. First, the number of patients was relatively small because bevacizumab and ramucirumab are not covered by Taiwan National Health Insurance program for lung cancer management. The small sample size prevents us to perform subsequent stratified analyses, such as those investigating the effects of EGFR mutation subtypes and the effectiveness of second‐line osimertinib on outcomes. A larger study is warranted for detailed stratified analyses. Second, our patients received bevacizumab at a dose of 7.5 mg/kg every 3 weeks, which was different from the dose adopted in the NEJ026 and JO25567 studies. Because a dose of 7.5 mg/kg was as effective as a dose of 15 mg/kg while being used in combination with chemotherapy, the bevacizumab dose of 7.5 mg/kg as an add‐on therapy was widely adopted in clinical practice worldwide.^38^ Third, only 64% of patients had T790M testing on disease progression during the first‐line therapy, as the feasibility of re‐biopsy was dependent on the recurrent tumor location and the patients' clinical condition. Fourth, in both univariate and multivariable analyses of overall survival, the hazard ratio of operation could not be estimated because all seven patients receiving operation remained alive at the end of our study period. Although patients underwent operation might have better OS, further studies with larger sample sizes and longer follow‐up periods are warranted.

In conclusion, our study demonstrated that the addition of bevacizumab or ramucirumab to an EGFR‐TKI as a front‐line treatment provided similar PFS and OS, while ramucirumab user had significantly high‐grade hypertension. Further large‐scale prospective studies are needed to confirm our findings.

## CONFLICT OF INTEREST STATEMENT

The authors declare no conflict of interest.
